# Evaluation of *BRCA1*-related molecular features and microRNAs as prognostic factors for triple negative breast cancers

**DOI:** 10.1186/s12885-015-1740-9

**Published:** 2015-10-21

**Authors:** Meriem Boukerroucha, Claire Josse, Sonia ElGuendi, Bouchra Boujemla, Pierre Frères, Raphaël Marée, Stephane Wenric, Karin Segers, Joelle Collignon, Guy Jerusalem, Vincent Bours

**Affiliations:** 1 Human Genetics Unit, GIGA-Cancer Research, University of Liège, Liège, Belgium; 2 Medical Oncology Department, University of Liège and CHU de Liège, Liège, Belgium; 3 Center of Genetics, CHU de Liège, Liège, Belgium; 4 GIGA-bioinformatics platform, University of Liège, Liège, Belgium

**Keywords:** *BRCA1*, TNBC, Breast cancer, miRNA

## Abstract

**Background:**

The *BRCA1* gene plays a key role in triple negative breast cancers (TNBCs), in which its expression can be lost by multiple mechanisms: germinal mutation followed by deletion of the second allele; negative regulation by promoter methylation; or miRNA-mediated silencing. This study aimed to establish a correlation among the *BRCA1*-related molecular parameters, tumor characteristics and clinical follow-up of patients to find new prognostic factors.

**Methods:**

BRCA1 protein and mRNA expression was quantified *in situ* in the TNBCs of 69 patients. *BRCA1* promoter methylation status was checked, as well as cytokeratin 5/6 expression. Maintenance of expressed BRCA1 protein interaction with BARD1 was quantified, as a marker of BRCA1 functionality, and the tumor expression profiles of 27 microRNAs were determined.

**Results:**

miR-548c-5p was emphasized as a new independent prognostic factor in TNBC. A combination of the tumoral expression of miR-548c and three other known prognostic parameters (tumor size, lymph node invasion and CK 5/6 expression status) allowed for relapse prediction by logistic regression with an area under the curve (AUC) = 0.96.

*BRCA1* mRNA and protein *in situ* expression, as well as the amount of BRCA1 ligated to BARD1 in the tumor, lacked any associations with patient outcomes, likely due to high intratumoral heterogeneity, and thus could not be used for clinical purposes.

**Conclusions:**

*In situ BRCA1*-related expression parameters could be used for clinical purposes at the time of diagnosis. In contrast, miR-548c-5p showed a promising potential as a prognostic factor in TNBC.

**Electronic supplementary material:**

The online version of this article (doi:10.1186/s12885-015-1740-9) contains supplementary material, which is available to authorized users.

## Background

Breast cancer susceptibility gene 1 (*BRCA1*) was the first tumor suppressor gene identified in breast and ovarian cancer. Located on chromosome 17 (17q21), it encodes a multifunctional protein that is involved in several cellular processes such as DNA repair and cell cycle control. BRCA1 is involved in large protein complexes and its interaction with other proteins, as BARD1, is required for its function.

*BRCA1* seems to be associated with the triple negative breast cancer (TNBC) subtype because the histological features and clinical outcomes of TNBC sporadic tumors can be very similar to those found in the tumors of *BRCA1* germline mutated patients. The traits that some sporadic cancers share with those occurring in *BRCA1* mutation carriers were described and called ‘BRCAness’ by Turner et al. [1]. In particular, these cancers present a high rate of chromosomal alterations reflecting the absence of the BRCA1 DNA repair function.

TNBC has a poor prognosis and no targeted therapy is currently available. Because these cancers are heterogeneous in terms of therapeutic response, new therapeutic solutions are being sought. In this context, recent clinical data have shown that *BRCA1*-associated breast cancers appeared to be more sensitive to platinum agents in neoadjuvant chemotherapy than non-hereditary tumors [2–4]. In contrast, a phase II clinical trial found that poly (ADP-ribose) polymerase (PARP) inhibitors also showed promising activity in *BRCA1*-mutated breast cancer although there were no response in patients with TNBC regardless of *BRCA1*/2 mutation status [5], and phase III trials are ongoing in *BRCA1*/2-mutated BC and TNBC [6].

However, it is also becoming clear that germline *BRCA1*/2 mutations are neither necessary nor sufficient for patients to derive benefit from these agents [6]. This variability of response can be explained by different BRCA1 protein expression statuses inside the tumor, as several cases can be met : (i) germline *BRCA1* is mutated in one allele, and the second is lost; thus, BRCA1 tumoral expression is missing [7, 8] (ii) germline *BRCA1* is mutated in one allele, and the second is still active, so tumoral BRCA1 expression is normal; (iii) germline *BRCA1* is mutated, but reversal somatic mutation occurs, and BRCA1 tumoral expression is restored, leading to PARP inhibitor treatment resistance [9, 10]; (iv) germline *BRCA1* is normal, but tumoral expression is lost by promoter hypermethylation [11]; and (v) germline *BRCA1* is normal, but tumoral expression is lost by post-transcriptional regulation, such as by miRNAs [12]. One could expect a greater likelihood of response for patients treated with platinum compounds or PARP inhibitors only if BRCA1 protein tumoral expression were lost. As a consequence, better characterization of BRCA1 expression status in TNBC would provide important knowledge to improve chemotherapy choices.

MicroRNAs are small non-coding RNAs that bind to the 3’ untranslated (3’UTR) region of target messenger RNAs (mRNAs), and they are known to regulate gene expression. They are deregulated in breast cancers: some of them are known to be oncogenic, and others are known as tumor suppressors. MiRNAs participate in a variety of biological processes, such as the immune response, as well as proliferation and metastasis, which are hallmarks of cancer [13, 14]. Many studies have implicated miRNAs in chemotherapy resistance, such as to cisplatin [15], and some of them have been used as prognostic biomarkers [16–18]. Moreover, some miRNAs could target BRCA1 mRNA expression, and, at the same time, their expression was affected by BRCA1 protein [12, 19–21].

Currently, the number of conventional breast cancer prognostic factors is limited (tumor size, histology and grade, hormone receptors status, lymph nodes invasion, proliferative index [Ki67], and tumor-infiltrating lymphocytes, as well as the age of the patient), and their use does not allow for accurate prediction of treatment resistance or relapse in TNBC. Defining new molecular prognostic factors to refine TNBC classification would be useful in facilitating a more adapted chemotherapy choice.

In this context, we quantified molecular parameters focusing on the *BRCA1* gene expression regulation and function (BRCA1 promoter methylation, BRCA1 *in situ* mRNA expression, BRCA1 *in situ* protein expression and BRCA1 *in situ* interaction with BARD1) in 69 TNBC tumors from patients. The expression of 27 tumoral miRNAs was also measured. Those molecular parameters were associated with progression-free survival in uni- and multivariate statistical analyses to determine new prognostic factors.

## Methods

More detailed protocols are available in Additional file 1.

### Ethical statement

Ethical approval was obtained from the local institutional ethics board (Comité d’éthique hospitalo-facultaire universitaire de Liège) in compliance with the Helsinki declaration. All of the patients were recruited on the basis of an opt-out methodology.

### Patient and sample collection and study design

This retrospective study was performed on 69 formalin-fixed paraffin embedded (FFPE) tumoral samples obtained from the Liege University Biobank. The tissues stored in this biobank are available on condition that the study has received the consent of a local or external ethical board. The tumors were collected from 1999 to 2010, with a median follow-up of 11 years. The essential elements of “Reporting recommendations for tumor marker prognostic studies (REMARK)” were followed [22].

The clinicopathological characteristics of the patients are summarized in Table 1.

**Table 1:** Patient clinicopathological characteristics

	*n* =*69*
*Age* (*year*)	
median	56
range	27–89
*Tumor size* (*mm*)	
< 20	23
≥ 20	35
unknown	11
*Lymph node invasion*	
yes	15
no	38
unknown	16
*Ki 67* (%)	
< 20	11
≥ 20	52
unknown	6
*Histology*	
IDC	47
other	19
unknown	3
*Bloom*	
I	6
II	7
III	53
unknown	3
*Molecular subtype*	
ck5/6 +, ER-, Her2-	30
ck5/6 -, ER-, Her2-	32
unknown	7
*Relapse*	
yes	24
no	45

**Fig. 1:**
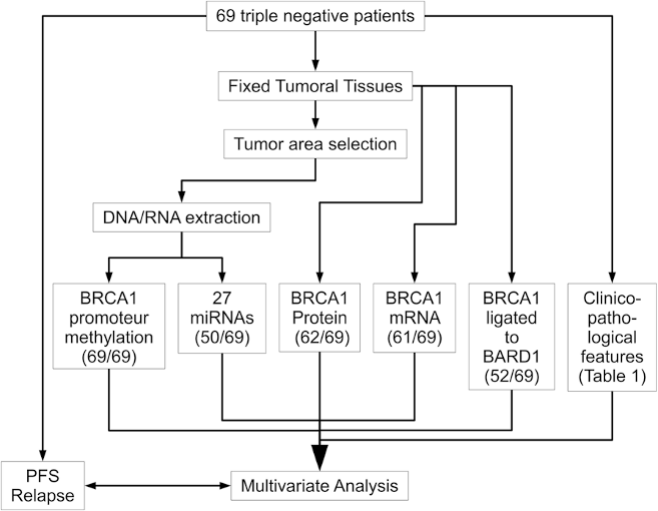
Schematic representation of the study

A summary of the experimental design and the number of samples included in each type of analysis are shown in Fig. 1.

### DNA and RNA extraction

DNA and RNA extraction was performed using an All Prep DNA/RNA FFPE extraction kit from Qiagen (Belgium) according to manufacturer protocol. Multiplex PCR for increasing the size amplicons of a house keeping gene was performed to assess the nucleic acid quality, as described by van Beers et al. [23].

### BRCA1 promoter methylation

The methylation status of *BRCA1* promoter was assessed by methylation-specific PCR (MSP), as described by Esteller et al. [24].

### BRCA1 mRNA expression

The mRNA expression was assessed by *in situ* hybridization using RNAscope technology (ACD) (Bioke, the Netherlands) for FFPE samples, as described in our previous work [25]. Signal quantification was performed using the Cytomine application (http://www.cytomine.be/, Marée et al. 2013) [26]. *BRCA1* mRNA expression was expressed as a percentage of the median expression value measured in the whole group.

#### BRCA1 protein expression and interaction with BARD1

BRCA1 expression level and interaction with BARD1 were assessed by proximity ligation assay (Duolink *in situ* detection reagents—Sigma, Belgium), as described in [25] and in Additional file 1. Two antibodies raised against BRCA1 were used for the whole-length protein detection assays, and one antibody against BRCA1 and a second against BARD1 were used for interaction assays. The amount of BRCA1 protein and the amount of BRCA1-ligated to BARD1 were expressed as a percentage of their respective median expression values measured in the whole group.

#### Tumoral miRNA expression assessment

A total of 27 miRNAs were quantified by RT-qPCR in tumors using miRCURY LNA™ Universal RT microRNA PCR assays from Exiqon (Denmark), according to the manufacturer’s instructions. Those miRNAs were chosen because: (i) their expression was reported in the literature to be related to the survival of breast cancer patients; (ii) they are known to be expressed in lymphoid cells and to reflect the lymphoid invasion of the tumor; or (iii) they were emphasized in our previous work (unpublished results). The miRNAs quantified, their sequences and the reasons for choosing them are listed in Additional file 2.

Quantification was realized using standard curve method. Normalization was performed using the geometric mean of five endogenous control genes. The miRNA amounts were expressed in percentages relative to the median expression value of the whole group.

#### Statistical analysis

Statistical analysis were performed with SPSS software (version 20.0: IBM SPSS), and checked with R software (version 3.1.0). Some of the graphs were drawn with GraphPad Prism software, version 5.

## Results

### Quantification of *in situ BRCA1* mRNA and protein expression

**Fig. 2:**
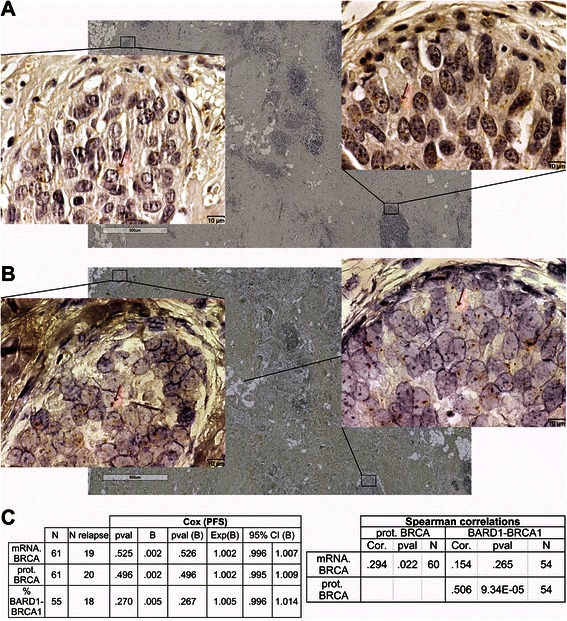
*In situ* BRCA1 expression in TNBC tumors. **a** Proximity ligation assay showing a representative BRCA1 protein expression across the tumor. Two different subzones were magnified to illustrate high and faint expression. **b** *In situ* hybridization assay showing *BRCA1* mRNA expression across the same tumor and subzones used for protein detection. In both cases, high heterogeneity of the localization of expression is observed. **c**. Cox univariate regression and correlation analyses of BRCA1 expression relative to patient clinicopathological features. No relationship of BRCA1 expression with patient outcome was observed

To assess the BRCA1 expression status inside the tumors, the amount of BRCA1 protein was first measured by proximity ligation assays (PLAs) in fixed TNBC tissues. Representative *in situ* BRCA1 protein expression is shown in Fig. 2a. As a second step, the *BRCA1* mRNA expression level was visualized and quantified in the same tissues, by *in situ* hybridization (Fig. 2b).

The most striking observation was that the staining for both mRNA and protein is heterogeneous across the tumor: some areas strongly expressed BRCA1 and others only faintly, as illustrated in the two magnified subzones. The staining was restricted to epithelial cells.

Univariate analyses showed that neither BRCA1 protein nor mRNA expression was associated with progression-free survival (PFS) (Fig. 2c). The entire dataset and all of the univariate analyses performed in this study are available in Additional files 3 and 4.

### Quantification of *in situ* BRCA1-BARD1 interaction

Proximity ligation assays were performed to quantify the *in situ* interaction of BRCA1 with its interacting protein, BARD1. Statistical analyses revealed that the percentage of BARD1-ligated BRCA1 was correlated with BRCA1 protein and mRNA expression. However, no association was observed with PFS in univariate analysis (Fig. 2c).

### *BRCA1* promoter methylation and survival

The methylation status of *BRCA1* promoter was checked by methylation-specific PCR in tumoral DNA extracted from fixed TNBC tissues. Twenty-seven of the 69 patients (39 %) carried a methylated *BRCA1* promoter, but we did not observe any associations of *BRCA1* promoter methylation with patient outcomes or with *BRCA1* mRNA expression (Additional file 5). However, an expected negative correlation was observed between methylation and protein expression in the infiltrating ductal carcinoma sub-group.

### Micro-RNA profiling in tumors

The tumoral expression of 27 miRNAs was quantified by RT-qPCR in RNA extracted from fixed TNBC tissues.

Spearman’s correlations were calculated of the studied miRNAs and *BRCA1* mRNA with protein expression, BARD1-BRCA1 interaction, and promoter methylation status. The entire dataset is presented in Additional file 3. BRCA1 protein expression was positively correlated with miR-143-3p (*p* = 0.033), miR-205-5p (*p* = 0.030), miR-21-5p (*p* = 0.017), and miR-142-5p (*p* = 0.011). In contrast, no correlation was noted with *BRCA1* mRNA. Promoter methylation was negatively correlated with miR-21-5p (*p* = 0.024) and positively correlated with miR-197-3p (*p* = 0.019).

Univariate Cox regression analyses were also conducted to emphasize the associations of miRNA expression with patient outcomes (Table 2 and Additional file 4). High expression of miR-210, miR-205-5p, miR-484, and miR-93-5p were significantly associated with an increased risk of relapse, and miR-342-3p, reflecting lymphoid cell infiltration [27], was associated with a good prognosis (Table 2).

**Table 2:** Univariate Cox analysis

*Variable*	*N total*	*N relapse*	*Global pval*	*B*	*Sign*	*Exp*(*B*)	*95* % *CI*	
miR-210	49	20	0.00	.004	.000	1.004	1.002	1.007
miR-205-5p	49	20	0.00	.003	.002	1.003	1.001	1.005
Node	53	21	0.00	−1.344	.003	.261	.108	.630
miR-484	49	20	0.01	.004	.015	1.004	1.001	1.008
CK	61	20	0.02	1.106	.024	3.023	1.159	7.881
miR-93-5p	49	20	0.02	.003	.019	1.003	1.001	1.006
Bloom = 3	65	23	0.03	1.963	.055	7.117	.957	52.955
miR-342-3p	49	20	0.04	−.005	.049	.995	.990	1.000
Size	57	18	0.05	.016	.055	1.016	1.000	1.032
Age	68	24	0.07	.025	.070	1.025	.998	1.053
Bloom = 1	65	23	0.08	−3.195	.262	.041	.000	10.892
miR-146a	49	20	0.09	−.004	.093	.996	.992	1.001
miR-143-3p	49	20	0.11	.003	.116	1.003	.999	1.007
miR-155-5p	49	20	0.11	−.003	.123	.997	.993	1.001
miR-150-5p	49	20	0.12	−.004	.151	.996	.991	1.001
miR-142-3p	49	20	0.18	−.003	.195	.997	.993	1.002
miR-548c-5p	49	20	0.19	−.001	.196	.999	.997	1.001
miR-374a-5p	49	20	0.20	−.005	.200	.995	.988	1.003

### Prediction of relapse using multivariate analysis

Univariate Cox proportional hazards regression analyses were first conducted to evaluate the association of clinicopathological factors with patient PFS (Additional file 4). Node invasion, cytokeratin five and six expression, bloom = 3 and the size of the tumor are associated with relapse.

In multivariate Cox analysis, three parameters remained as independent prognostic factors: node invasion status, tumor size, and the expression of miR-548c-5p (node invasion: Exp(B): 16.576; CI: 2.876–95.538; *p*-val: 0.002—tumor size: Exp(B): 1.065; CI: 1.027–1.105; *p*-val: 0.001—miR-548c-5p: Exp(B): 0.993; CI: 0.987–0.999; *p*-val: 0.023).

**Fig. 3:**
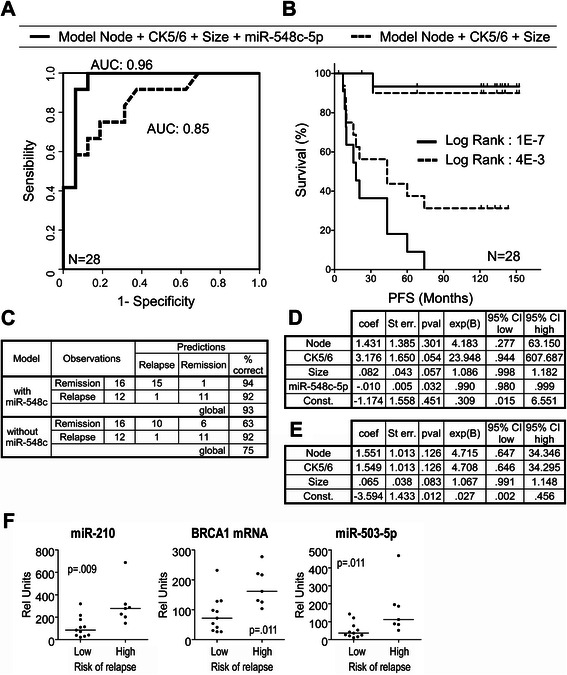
miR-548c-5p as factor in relapse prediction model. Performances of two models are compared to measure the improvement of relapse prediction by the inclusion of the miR-548c-5p as a 4th variable, with the first three variables being node invasion, CK5/6 expression, and tumor size. **a** Comparison of ROC curves computed with the relapse probability calculated by the model including miR-548c-5p (*solid line*) and without miR-548c-5p (*dash line*). **b** Patients were classified in two groups: high and low risk of relapse, according to the threshold needed to obtain 90 % sensitivity in relapse prediction. Comparison of Kaplan-Meier curves computed with the patient group affectation calculated by the model including miR-548c-5p (*solid line*) and without miR-548c-5p (*dash line*). **c** Classification performances of the two models at thresholds fixed to obtain 90 % sensitivity in relapse detection. **d** Coefficient and odds ratio of the model including miR-548c-5p and **e** without miR-548c-5p. **f** Comparative expression levels of miR-210, miR-503-5p and *BRCA1* mRNA in the patients with <10 % probability of relapse (remission) and >90 % probability of relapse (relapse). These probabilities were calculated by the prediction model including miR-548c-5p

An outcome prediction model was built by binomial logistic regression. The best prediction model used node invasion, the size of the tumor, cytokeratin 5/6 expression status and miR-548c-5p. Because the first three variables were already known to be prognostic factors, we compared the performances of two models, containing or not containing the miR-548c-5p expression variable, to evaluate the improvement of the prediction of relapse by this miRNA (Fig. 3). The addition of miR-548c-5p statistically improved the model (Chi-square *p*-val = 0.00144). A ROC curve corresponding to the probability of relapse for each patient, calculated by these two models, is shown in Fig. 3a. The use of miR-548c-5p expression allowed for the improvement of the AUC from 0.854 (CI:0.713 to 0.996) to 0.958 (CI:0.883–1.000)(Table 3). Thresholds for both models were chosen to fix relapse detection sensitivity at 90 %. Using these thresholds, the patients were assigned by each model into two risk groups: low or high risk of relapse. Kaplan-Meier PFS curves were generated using these effect groups for both models, and they are shown in Fig. 3b. Classification performances of the compared models are presented in Fig. 3c, and metrics are shown in Fig. 3d (with miR-548c) and 3e (without miR-548c) and in Table 3.

**Table 3:** Performances metrics of the logistical regression models

	*Global model performances*			*ROC*				*Hosmer*-*Lemeshow*		
*Variables*	*pval*	−*2LL*	*R2 Nagelkerke*	*AUC*	*SE*	*CI*: *Min*	*CI* : *Max*	*Chi2*	*ddl*	*pval*
Node, Tumor Size, CK5/6, miR-548c-5p	1.5E-4	15.570	.745	.96	.038	.883	1.000	5.08	7	.65
Node, Tumor Size, CK5/6, prot BRCA1, mRNA BRCA1, BARD1 ligated BRCA1	.009	21.979	.590	.90	.065	.773	1.000	11.45	8	.18
Node, Tumor Size, CK5/6	.006	25.722	.484	.85	.072	.713	.996	2.96	7	.89

Interestingly, a comparison of two groups of patients presenting with extreme relapse probabilities (<10 % and >90 %), calculated by the predicting model including miR-548c, showed that patients with poor prognoses present higher expression of miR-503-5p, miR-210 and *BRCA1* mRNA.

**Fig. 4:**
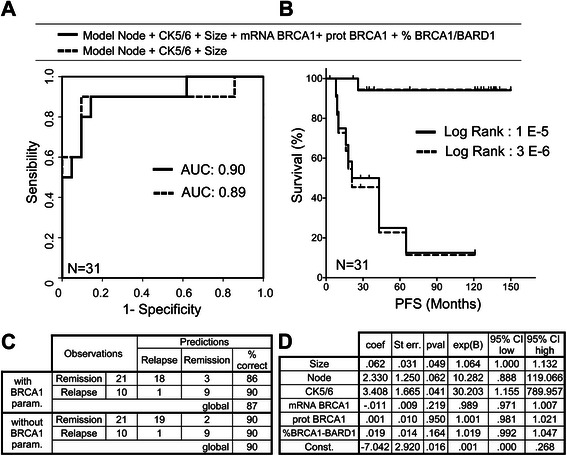
BRCA1 expression as factor in relapse prediction models. Performances of two models are compared: the first model (solid line) includes BRCA1 expression parameters mRNA, protein expression and BARD1-ligated BRCA1, in addition to the three previously used conventional prognostic factors for breast cancer: tumor size, node invasion, CK5/6 expression. The second model (*dash line*) is composed of the three conventional prognostic factors only. **a** Comparison of ROC curves computed with the relapse probability calculated by the BRCA1-related model (solid line) and the three conventional prognostic factor model (*dash line*). **b** Patients were classified in two groups: good or bad prognosis, according to the threshold needed to obtain 90 % sensitivity in relapse detection. Comparison of Kaplan-Meier curves computed with the patient group affectation calculated by the two models is represented. **c**. Classification performances of the two models at threshold fixed to obtain 90 % sensitivity in relapse detection. **d**. Coefficient and odds ratio of the model including BRCA1 expression parameters

In contrast, the addition of *BRCA1*-related parameters (mRNA, protein and BARD1 ligated to BRCA1) to the same three conventional prognostic factors (node invasion, tumor size and CK5/6 expression) did not improve the model performances (Fig. 4 and Table 3).

## Discussion

An accurate technique to determine BRCA1 tumoral expression status in TNBC would allow for informed decision and choosing platinum derivatives or PARP inhibitor treatments, because hypersensitivity to these agents has been described in cases of loss of BRCA1 expression [3, 5]. Thus, we developed alternative techniques to evaluate, in tumors, the expression status of BRCA1 at three different levels: mRNA, protein, and maintenance of BRCA1 interaction with BARD1. This multiple approach presented the advantage of incorporating different types of information, allowing for cross-control, and offering the possibility of combining the data. Several *BRCA1* studies have described mRNA expression using RT-qPCR or protein expression by immunohistochemistry, but studies describing both mRNA and protein expressions has been very rare [28], despite BRCA1 expression being known to be subjected to multiple regulations [19]. The commercially available antibodies directed against BRCA1 lack the specificity required to identify the BRCA1 protein for clinical purpose because no immunohistochemical (IHC) differences in BRCA1 protein expression were found between cases with and without *BRCA1* germline mutations by Pérez-Vallés and colleagues [29]. To improve the sensitivity and specificity of the BRCA1 detection compared with IHC, we used proximity ligation assays with two primary antibodies against the N- and C-terminus domains of BRCA1. The second advantage of this technique was that it only allowed for the measurement of the full-length proteins. BRCA1 must be ligated to its interacting protein BARD1 to repair DNA. Some *BRCA1* variants, such as splicing variants [29], can be expressed in the tumor but can lose their interaction with their partners. To obtain a reflection of BRCA1 function maintenance in tumors, proximity ligation assay were performed to visualize the portion of BRCA1 ligated to BARD1.

Although the three levels of BRCA1 tumoral expression were correlated inside the same tumor, highly heterogenous intra-tumoral expression was observed, hampering accurate quantification. The lack of correlation between PFS and BRCA1 expression was probably a consequence of this high variability. We concluded that none of these three facets of the BRCA1 tumoral expression could be used for clinical decision purposes.

The TNBC cohort that we explored included six patients with a known germline *BRCA1* mutation. However, no significant differences in BRCA1 expression at the levels of mRNA, protein, or ligation to BARD1 were observed in these cases, probably due to the small number of patients. Interestingly, two of these six *BRCA1* mutated patients also presented a methylated form of the *BRCA1* promoter, although Lips et al. described these events as mutually exclusive [30]. This combination of events could increase the risk of breast cancer because these patients are also the two youngest who developed breast cancer in our cohort of 69 patients, but this possibility will need to be confirmed on a larger cohort. The work of Ertuk and Cecener also stated that miRNAs expression can be different in *BRCA1* mutated or normal TNBC tumors [31]. However, we could not observe similar effect, probably due to the small number of patients.

Statistical multivariate analysis demonstrated that miR-548c-5p was an independent prognostic factor for breast cancer. Patients with a good prognosis presented higher intratumoral expression of this microRNA. Although implicated in multiple biological processes including cancer, no role for miR-548c-5p has ever been reported in the breast cancer field. Mir-548 is a large, poorly conserved primate-specific miRNA gene family. Sixty-nine human mir-548 genes are located on almost all human chromosomes and its widespread distribution pattern and specific sequence indicate its evolutionary origin from the MADE1 transposable element [32, 33]. There are more than 3500 putative mir-548 target genes, but none have been experimentally demonstrated for miR-548c-5p.

The measurement of tumoral miR-548c-5p expression levels in combination with three conventional breast cancer prognostic factors (node invasion, tumor size and cytokeratin 5/6 expression), allowed for the relapse prediction of patients with an AUC = 0.96. A study in a larger cohort would be needed to confirm this observation, and to determine whether quantification of this microRNA expression in the tumor could be used to steer patients with poor predicted prognosis toward alternative chemotherapies.

We also showed that patients with poor predicted prognoses calculated by this model presented higher expression of miR-210, miR-503-5p and *BRCA1* mRNA. Indeed, high miR-210 expression has already been reported by other teams to be correlated with relapse and short survival [19, 34]. miR-503-5p was already emphasized in our previous work: this microRNA is highly expressed in endothelial cells and, can be secreted in exosomes and transferred into breast cancer cell lines to inhibit tumor growth by targeting CCND2 and CCND3 [35]. Moreover, neoadjuvant chemotherapy for breast cancer leads to increased plasma levels of miR-503, as also observed for miR-34a, which could be implicated in the anti-tumor effects of chemotherapy in breast cancer patients [35, 36]. Concerning the higher expression of *BRCA1* mRNA observed in the poor-prognosis tumors, we could hypothesize that patients expressing high levels of *BRCA1* would present a lower response to chemotherapy because TNBC *BRCA1* mutated patients are known to respond better to chemotherapy [37].

MiR-484 was reported by Dvinge et al. as a good potential housekeeping microRNA in breast cancer because its expression was homogenous among samples in all breast cancers subtypes [27]. However, Cox univariate analysis showed that high miR-484 expression was associated with a bad prognosis in our TNBC cohort. Volinia et al. also reported such an association [18]. In a high-throughput study aiming at better defining miRNA-mRNA interaction, *BRCA1* was identified as interacting with miR-484. However, we did not observed any inverse correlation between those two parameters. Although, miR-484 expression was strongly associated with two other poor-prognosis miRNAs: miR-205 (Rho Spearman: 0.4, *p*-val :0.003) and miR-93 (Rho Spearman : 0.52, *p*-val = 0.0001), the Diana MiRPath database did not present any experimentally demonstrated common target gene of the three miRNAs [38].

## Conclusions

BRCA1 was expressed in a spatially heterogeneous manner in TNBC, making very difficult any study correlating its expression or activity with prognosis. However, this study emphasized miR-548c-5p tumoral expression as a new independent prognostic factor that could improve the performance of relapse prediction models based on node invasion, tumor size and cytokeratin five and six expression status.

## Additional files

Additional file 1: **Detailed description of the protocols used in the study.** (DOCX 24 kb)
Additional file 2: **List of the microRNAs quantified by RT-qPCR, their sequences and the reasons for their choice for the study.** The most significant MSigDB Canonical Pathways affected by each microRNAs are mentioned as provided by the StarBase v2.0 web server issue [39]. (DOCX 58 kb)
Additional file 3: **Table of the entire variable and observation dataset.** (XLS 53 kb)
Additional file 4: **Results of the univariate analyses performed on the dataset.** (XLS 109 kb)
Additional file 5: ***BRCA1*** **promoter methylation.** A. Kaplan Meier curves showing the lack of relationship of BRCA1 promoter methylation with progression-free survival. B. Cox univariate regression and correlation analyses of *BRCA1* promoter methylation relative to patient clinicopathological features. (TIFF 307 kb)
