# Supplementary material for: Evaluation of BRCA1-related molecular features and microRNAs as prognostic factors for triple negative breast cancers
Source: BMC Cancer. 2015 Oct 21;15:755. doi: 10.1186/s12885-015-1740-9 (PMC4618357; doi:10.1186/s12885-015-1740-9)
Supplement: Additional file 1: — Detailed description of the protocols used in the study. (DOCX 24 kb) [file 12885_2015_1740_MOESM1_ESM.docx]

**Additional materiel and methods:**

**Patients and samples collection:**

69 formalin (10%) fixed paraffin embedded (FFPE) triple negative breast tumors were collected from 1999 to 2010, with a median follow-up of 11 years.

4 µM tissue sections were mounted on slide glass and haematoxylin eosin stained. Twenty additional slides were used for nucleic acids extraction.

***DNA and RNA isolation:***

In order to maximize tumoral content, one tumor haematoxylin-eosin stained section was examined by a pathologist to determine the tumor area. To isolate nucleic acids, the annotated stained section was superposed with 19 unstained sections, the tumor area was manually macro-dissected with sterile needle and collected in a 2ml tube.

***BRCA1 Promoter methylation:***

250 ng DNA were subjected to sodium bisulfite conversion using EZ DNA Methylation kit according to the manufacturer’s instructions. Methylation status of BRCA1 promoter was assessed by methylation specific PCR (MSP) using HotStarTaq DNA polymerase (Qiagen, Belgium). PCR was performed in a Verity thermal cycler (Applied Biosystem, Belgium) as follow: 15 min 95°C; 35 cycles of 30 sec 94°C, 30 sec 58°C and 45sec 72°C; followed by 10 min 72°C. Sequences of the primers are BRCA1-METHYL R (5'-AAATCTCAACGAACTCACGCCG-3'); BRCA1-METHYL F (5'-TCGTGGTAACGGAAAAGCGC-3'); BRCA1-UNMETH R (5'-CAAAAAATCTCAACAAACTCACACCA-3'); BRCA1-UNMETH F (5'-TTGGTTTTTGTGGTAATGGAAAAGTGT-3').

***BRCA1 mRNA expression*:**

The BRCA1 probe used is complementary to mRNA coding for aa369-1482, spanning exon 5 to exon 11. Whole slide scanned images of the BRCA1 staining were transferred to an in-house developed application called Cytomine to quantify signal (http//www.cytomine.be/, Marée *et al.* 2013). Spots and cells were independently and blindly counted twice, in several subzones of the tumor. The ratio between the number of spots and the number of cells was calculated and compared to the median of the whole group.

Marée R, Stévens B, Rollus L, et al. (2013) A rich internet application for remote visualization and collaborative annotation of digital slides in histology and cytology. Diagn Pathol 8:S26. doi: 10.1186/1746-1596-8-S1-S26

***BRCA1 protein expression:***

The protein level expression was assessed by proximity ligation assay (Duolink *in situ* detection reagents – Sigma, Belgium) according to the manufacturer's instructions. Two primary antibodies against the N-terminus and the C-terminus domain of BRCA1 were used for detection, improving the sensitivity and the specificity of the reaction as compared to IHC, and allowing the only detection of the BRCA1 full-length proteins.

The primary antibodies are raised in different species and are recognized by two secondary antibodies coupled with oligonucleotide probes. After ligation of the two probes, the circular DNA is amplified by polymerase reaction. The detection is performed using horseradish peroxidase (HRP) labeled probes and a chromogenic reaction using 3,3’Diaminobenzidine (DAB). In order to generate a signal only with the full-length BRCA1 protein, the two primary antibodies where chosen to be specific to the N-term ([MS110] ab16780, Abcam) and C-term domain (Sigma, SAB4502848) of the BRCA1 protein, respectively. The protein expression level was estimated using the same quantification method as for mRNA expression level.

***BRCA1-BARD1 interaction:***

The *BRCA1-BARD1* proteins interaction was assessed by proximity ligation assay as described above. Primary antibodies used were purchased at Sigma Belgium (*BARD1*: WH0000580M1, *BRCA1*: SAB4502848). The BRCA1-BARD1 interaction was estimated using the same quantification method as for mRNA and BRCA1 protein expression levels.

**Tumoral miRNA expression assessment.**

FFPE-extracted nucleic acids quality test : After reverse transcription, two dilutions of cDNA of each patient were tested for amplification with primers for two endogenous control genes in order to check for cDNA integrity and to choose appropriate dilution for quantification. This quality check experiment leads to the elimination of 19/69 patients, probably due to poor total RNA quality extracted from FFPE tissues.

Quantification was realized using standard curve method, checking if Ct obtained were enclosed in the qPCR linearity range. Normalization was performed using the geometric mean of 5 endogenous control genes as recommended by Mestdagh et al (2009) (SNORD44, SNORD66, hsa-miR-190a-5p, hsa-miR-361-3p, hsa-miR-484) [14]. SNORD44 and SNORD66 are broadly used endogenous controls for miRNA quantifications. Hsa-miR-190a-5p, hsa-miR-361-3p and hsa-miR-484 were chosen on the base of their expression data published by Farazi et al (2011) , as they had a stable expression across breast tumours. Later, Dvinge et al (2013) confirmed that miR-484 expression can be used as endogenous control in breast cancer tissues. Relative miRNA expression of each of the 50 patients was obtained by comparison to a pool of cDNA from those patients. This pool was used to perform standard curves used for quantification.

Ref :

Farazi T a, Horlings HM, Ten Hoeve JJ, et al. (2011) MicroRNA sequence and expression analysis in breast tumors by deep sequencing. Cancer Res 71:4443–53. doi: 10.1158/0008-5472.CAN-11-0608

Dvinge H, Git A, Gräf S, et al. (2013) The shaping and functional consequences of the microRNA landscape in breast cancer. Nature 497:378–82. doi: 10.1038/nature12108umors by deep sequencing. Cancer Res 71:4443–53. doi: 10.1158/0008-5472.CAN-11-0608

**Statistical analysis**

All univariate statistical tests were two sided. Because of non-normal distribution of microRNA, Mann-Withney U test were performed.

The 17 variables to be included in the multivariate analysis were chosen with global pval of univariate Cox regression <0.2. (Table 2).

The best logistic regression and Cox regression multivariate models were constructed by ascending forward logistic regression analysis. A likelihood test was done to assess whether addition of each new variable in the logistic regression model gave a better fit. Receiver-operating-characteristic (ROC) curves were plotted and asymptotic confidence intervals calculated (CI), and the area under the ROC curve (AUC) was compared for each model. Hosmer-Lemeshow test was applied for assessing the optimism of the prediction rule. Kaplan-Meier curves were produced for the progression free survival (PFS) and overall survival (OS) of patients stratified by the prediction model. The P values of the survival differences in the Kaplan-Meier analyses were calculated by the log-rank test. Collinearity of all variables used together in logistic regression were checked for variance inflation factor (VIF) <3.
