# Supplementary material for: Evaluation of BRCA1-related molecular features and microRNAs as prognostic factors for triple negative breast cancers
Source: BMC Cancer. 2015 Oct 21;15:755. doi: 10.1186/s12885-015-1740-9 (PMC4618357; doi:10.1186/s12885-015-1740-9)
Supplement: Additional file 2: — List of the microRNAs quantified by RT-qPCR, their sequences and the reasons for their choice for the study. The most significant MSigDB Canonical Pathways affected by each microRNAs are mentioned as provided by the StarBase v2.0 web server issue [39]. (DOCX 58 kb) [file 12885_2015_1740_MOESM2_ESM.docx]

| miRNA name | MiR sequence | accession number | Reason of choice | MSigDB All Canonical Pathways [1] |
| --- | --- | --- | --- | --- |
| hsa-let-7b-5p | UGAGGUAGUAGGUUGUGUGGUU | MIMAT0000063 | [2–4] | TNFR2 Signaling Pathway |
| hsa-miR-128-3p | UCACAGUGAACCGGUCUCUUU | MIMAT0000424 | [5, 6] | RXR and RAR heterodimerization with other nuclear receptor |
| hsa-miR-135a-5p | UAUGGCUUUUUAUUCCUAUGUGA | MIMAT0000428 | [5] | ErbB signaling pathway |
| hsa-miR-150-5p | UCUCCCAACCCUUGUACCAGUG | MIMAT0000451 | [5], lymphoïd cell expression | JNK MAPK Pathway |
| hsa-miR-155-5p | UUAAUGCUAAUCGUGAUAGGGGU | MIMAT0000646 | [7–9], lymphoïd cell expression | IL4-mediated signaling events |
| hsa-miR-197-3p | UUCACCACCUUCUCCACCCAGC | MIMAT0000227 | [3] | Adherens junction |
| hsa-miR-21-5p | UAGCUUAUCAGACUGAUGUUGA | MIMAT0000076 | [3, 10, 11] | TGF-beta receptor signaling |
| hsa-miR-210-3p | CUGUGCGUGUGACAGCGGCUGA | MIMAT0000267 | [3, 5, 6] | Regulation of Telomerase |
| hsa-miR-27b-3p | UUCACAGUGGCUAAGUUCUGC | MIMAT0000419 | [5, 12] | Trk receptor signaling mediated by PI3K and PLC-gamma |
| hsa-miR-342-3p | UCUCACACAGAAAUCGCACCCGU | MIMAT0000753 | [5, 13], lymphoïd cell expression | Genes involved in Myogenesis |
| hsa-miR-34a-5p | UGGCAGUGUCUUAGCUGGUUGU | MIMAT0000255 | [9, 14] | Notch signaling pathway |
| hsa-miR-374a-5p | UUAUAAUACAACCUGAUAAGUG | MIMAT0000727 | [8, 13, 15, 16] | Regulation of nuclear SMAD2/3 signaling |
| hsa-miR-142-5p | CAUAAAGUAGAAAGCACUACU | MIMAT0000433 | [13], lymphoïd cell expression | / |
| hsa-miR-142-3p | UGUAGUGUUUCCUACUUUAUGGA | MIMAT0000434 | lymphoïd cell expression | Genes involved in Signaling by EGFR in Cancer |
| has-miR-342-5p | AGGGGUGCUAUCUGUGAUUGA | MIMAT0004694 | [13], lymphoïd cell expression | / |
| hsa-miR-146a-5p | UGAGAACUGAAUUCCAUGGGUU | MIMAT0000449 | lymphoïd cell expression | Genes involved in p75NTR recruits signalling complexes |
| hsa-miR-223-3p | UGUCAGUUUGUCAAAUACCCCA | MIMAT0000280 | lymphoïd cell expression | FoxO family signaling |
| hsa-miR-503-5p | UAGCAGCGGGAACAGUUCUGCAG | MIMAT0002874 | unpublished results | Regulation of eIF4e and p70 S6 Kinase |
| hsa-miR-23a-3p | AUCACAUUGCCAGGGAUUUCC | MIMAT0000078 | unpublished results | MAPKinase Signaling Pathway |
| hsa-miR-301b | CAGUGCAAUGAUAUUGUCAAAGC | MIMAT0004958 | [17], unpublished results | TGF-beta signaling pathway |
| hsa-miR-143-3p | UGAGAUGAAGCACUGUAGCUC | MIMAT0000435 | [17], unpublished results | Genes involved in NGF signalling via TRKA from the plasma membrane |
| hsa-miR-548c-5p | AAAAGUAAUUGCGGUUUUUGCC | MIMAT0004806 | unpublished results | / |
| hsa-miR-148b-3p | UCAGUGCAUCACAGAACUUUGU | MIMAT0000759 | [13] | Genes involved in Developmental Biology |
| hsa-miR-505-3p | CGUCAACACUUGCUGGUUUCCU | MIMAT0002876 | [2] | Genes involved in Developmental Biology |
| hsa-miR-93-5p | CAAAGUGCUGUUCGUGCAGGUAG | MIMAT0000093 | [18, 19] | Genes involved in G1 Phase |
| hsa-miR-205-5p | UCCUUCAUUCCACCGGAGUCUG | MIMAT0000266 | [4, 19, 20] | Genes involved in YAP1- and WWTR1 (TAZ)-stimulated gene expression |
| hsa-miR-378a-3p | ACUGGACUUGGAGUCAGAAGG | MIMAT0000732 | [3] | Genes involved in Circadian Clock |
| SNORD44 |  |  | endogenous control, snoRNA |  |
| SNORD66 |  |  | endogenous control, snoRNA |  |
| hsa-miR-190a-5p | UGAUAUGUUUGAUAUAUUAGGU | MIMAT0000458 | miRNA used as endogenous control | Genes involved in Regulatory RNA pathways |
| hsa-miR-361-3p | UCCCCCAGGUGUGAUUCUGAUUU | MIMAT0004682 | miRNA used as endogenous control | / |
| hsa-miR-484 | UCAGGCUCAGUCCCCUCCCGAU | MIMAT0002174 | miRNA used as endogenous control | / |

1. Li JH, Liu S, Zhou H, Qu LH, Yang JH: **StarBase v2.0: Decoding miRNA-ceRNA, miRNA-ncRNA and protein-RNA interaction networks from large-scale CLIP-Seq data**. *Nucleic Acids Res* 2014, **42**:92–97.

2. Jonsdottir K, Janssen SR, Da Rosa FC, Gudlaugsson E, Skaland I, Baak JP a, Janssen E a M: **Validation of expression patterns for nine miRNAs in 204 lymph-node negative breast cancers.** *PLoS One* 2012, **7**:e48692.

3. Volinia S, Galasso M, Sana ME, Wise TF, Palatini J, Huebner K, Croce CM: **Breast cancer signatures for invasiveness and prognosis defined by deep sequencing of microRNA.** *Proc Natl Acad Sci U S A* 2012, **109**:3024–9.

4. Quesne J Le, Jones J, Warren J, Dawson S-J, Ali HR, Bardwell H, Blows F, Pharoah P, Caldas C: **Biological and prognostic associations of miR-205 and let-7b in breast cancer revealed by in situ hybridization analysis of micro-RNA expression in arrays of archival tumour tissue.** *J Pathol* 2012, **227**:306–14.

5. Buffa FM, Camps C, Winchester L, Snell CE, Gee HE, Sheldon H, Taylor M, Harris AL, Ragoussis J: **microRNA-associated progression pathways and potential therapeutic targets identified by integrated mRNA and microRNA expression profiling in breast cancer.** *Cancer Res* 2011, **71**:5635–45.

6. Foekens J a, Sieuwerts AM, Smid M, Look MP, de Weerd V, Boersma AWM, Klijn JGM, Wiemer E a C, Martens JWM: **Four miRNAs associated with aggressiveness of lymph node-negative, estrogen receptor-positive human breast cancer.** *Proc Natl Acad Sci U S A* 2008, **105**:13021–6.

7. Gasparini P, Lovat F, Fassan M, Casadei L, Cascione L, Jacob NK, Carasi S, Palmieri D, Costinean S, Shapiro CL, Huebner K, Croce CM: **Protective role of miR-155 in breast cancer through RAD51 targeting impairs homologous recombination after irradiation**. *Proc Natl Acad Sci* 2014:1–6.

8. Cascione L, Gasparini P, Lovat F, Carasi S, Pulvirenti A, Ferro A, Alder H, He G, Vecchione A, Croce CM, Shapiro CL, Huebner K: **Integrated microRNA and mRNA signatures associated with survival in triple negative breast cancer.** *PLoS One* 2013, **8**:e55910.

9. Roth C, Rack B, Müller V, Janni W, Pantel K, Schwarzenbach H: **Circulating microRNAs as blood-based markers for patients with primary and metastatic breast cancer.** *Breast Cancer Res* 2010, **12**:R90.

10. Müller V, Gade S, Steinbach B, Loibl S, von Minckwitz G, Untch M, Schwedler K, Lübbe K, Schem C, Fasching P a, Mau C, Pantel K, Schwarzenbach H: **Changes in serum levels of miR-21, miR-210, and miR-373 in HER2-positive breast cancer patients undergoing neoadjuvant therapy: a translational research project within the Geparquinto trial.** *Breast Cancer Res Treat* 2014.

11. Markou A, Yousef GM, Stathopoulos E, Georgoulias V, Lianidou E: **Prognostic Significance of Metastasis-Related microRNAs in Early Breast Cancer Patients with a Long Follow-up**. *Clin Chem* 2013.

12. Tang W, Zhu J, Su S, Wu W, Liu Q, Su F, Yu F: **MiR-27 as a prognostic marker for breast cancer progression and patient survival.** *PLoS One* 2012, **7**:e51702.

13. Cimino D, De Pittà C, Orso F, Zampini M, Casara S, Penna E, Quaglino E, Forni M, Damasco C, Pinatel E, Ponzone R, Romualdi C, Brisken C, De Bortoli M, Biglia N, Provero P, Lanfranchi G, Taverna D: **miR148b is a major coordinator of breast cancer progression in a relapse-associated microRNA signature by targeting ITGA5, ROCK1, PIK3CA, NRAS, and CSF1.** *FASEB J* 2013, **27**:1223–35.

14. Svoboda M, Sana J, Redova M, Navratil J, Palacova M, Fabian P, Slaby O, Vyzula R: **MiR-34b is associated with clinical outcome in triple-negative breast cancer patients.** *Diagn Pathol* 2012, **7**:31.

15. Li J-Y, Zhang Y, Zhang W-H, Jia S, Kang Y, Tian R: **Effects of Differential Distribution of Microvessel Density, Possibly Regulated by miR-374a, on Breast Cancer Prognosis.** *Asian Pac J Cancer Prev* 2013, **14**:1715–20.

16. Cai J, Guan H, Fang L, Yang Y, Zhu X, Yuan J, Wu J, Li M: **MicroRNA-374a activates Wnt/β-catenin signaling to promote breast cancer metastasis.** *J Clin Invest* 2013, **123**:566–79.

17. Chang Y-Y, Kuo W-H, Hung J-H, Lee C-Y, Lee Y-H, Chang Y-C, Lin W-C, Shen C-Y, Huang C-S, Hsieh F-J, Lai L-C, Tsai M-H, Chang K-J, Chuang EY: **Deregulated microRNAs in triple-negative breast cancer revealed by deep sequencing.** *Mol Cancer* 2015, **14**:36.

18. Sempere LF, Christensen M, Silahtaroglu A, Bak M, Heath C V, Schwartz G, Wells W, Kauppinen S, Cole CN: **Altered MicroRNA expression confined to specific epithelial cell subpopulations in breast cancer.** *Cancer Res* 2007, **67**:11612–20.

19. Madden SF, Clarke C, Aherne ST, Gaule P, O’Donovan N, Crown J, Clynes M, Gallagher WM: **BreastMark: an integrated approach to mining publicly available transcriptomic datasets relating to breast cancer outcome.** *Breast Cancer Res* 2013, **15**:R52.

20. Blenkiron C, Goldstein LD, Thorne NP, Spiteri I, Chin S, Dunning MJ, Barbosa-Morais NL, Teschendorff AE, Green AR, Ellis IO, Tavaré S, Caldas C, Miska EA: **MicroRNA expression profiling of human breast cancer identifies new markers of tumor subtype.** *Genome Biol* 2007, **8**:R214.
