# Supplementary figures and images for: Evaluation of BRCA1-related molecular features and microRNAs as prognostic factors for triple negative breast cancers
Source: BMC Cancer. 2015 Oct 21;15:755. doi: 10.1186/s12885-015-1740-9 (PMC4618357; doi:10.1186/s12885-015-1740-9)

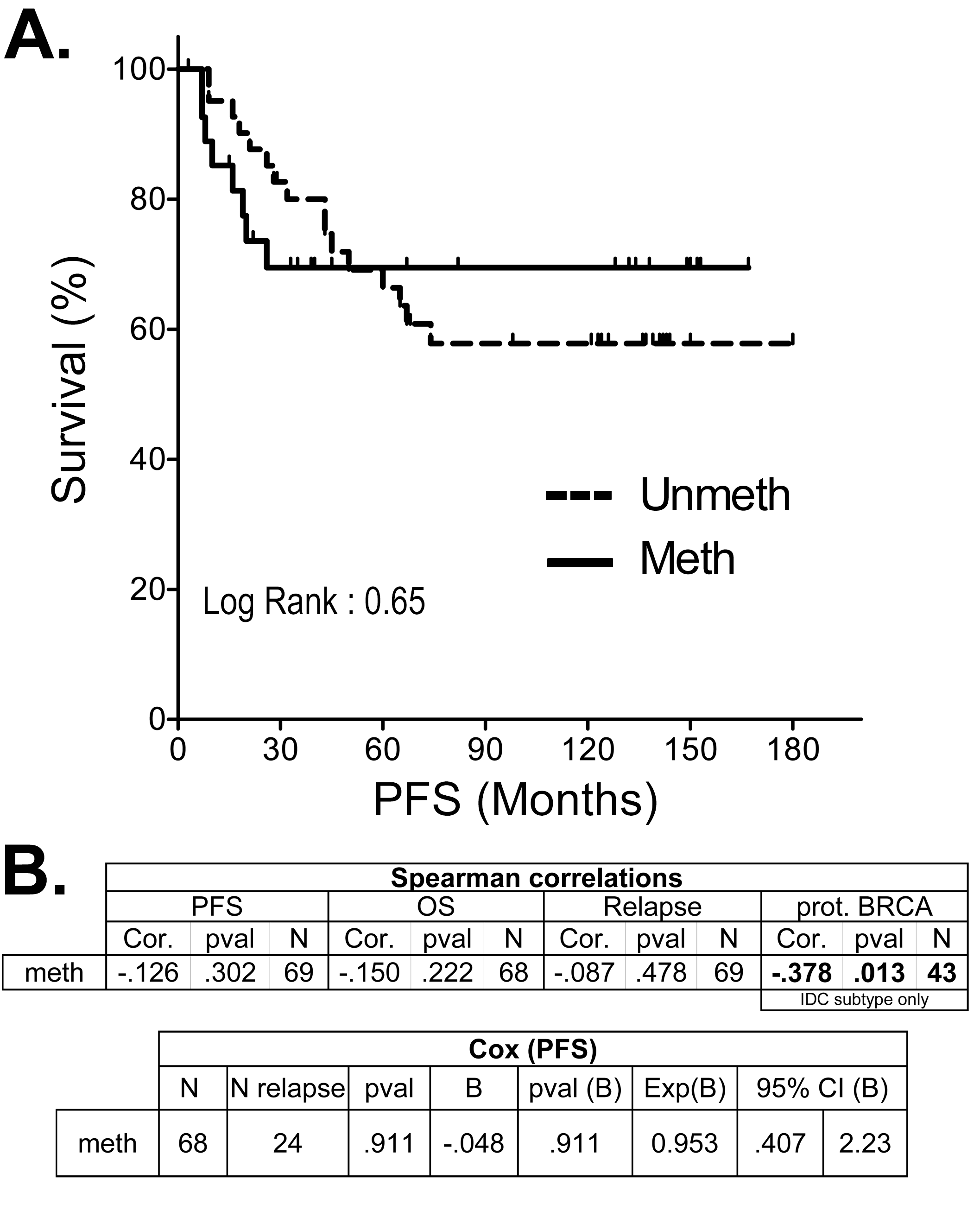

Supplement: Additional file 5: — BRCA1 promoter methylation. A. Kaplan Meier curves showing the lack of relationship of BRCA1 promoter methylation with progression-free survival. B. Cox univariate regression and correlation analyses of BRCA1 promoter methylation relative to patient clinicopathological features. (TIFF 307 kb) [file 12885_2015_1740_MOESM5_ESM.tiff]
